# Supplementary material for: Optimization of differentiation and transcriptomic profile of THP-1 cells into macrophage by PMA
Source: PLoS One. 2023 Jul 17;18(7):e0286056. doi: 10.1371/journal.pone.0286056 (PMC10351730; doi:10.1371/journal.pone.0286056)
Supplement: S1 File — (ZIP) [file pone.0286056.s001.zip › supporting files/RNA-seq report.zip!/RNA-seq report/5_KEGGEnrichment_result/induce_vs_control/Enrich_plot/induce_vs_control.DEG_KEGG_enriched.pdf]

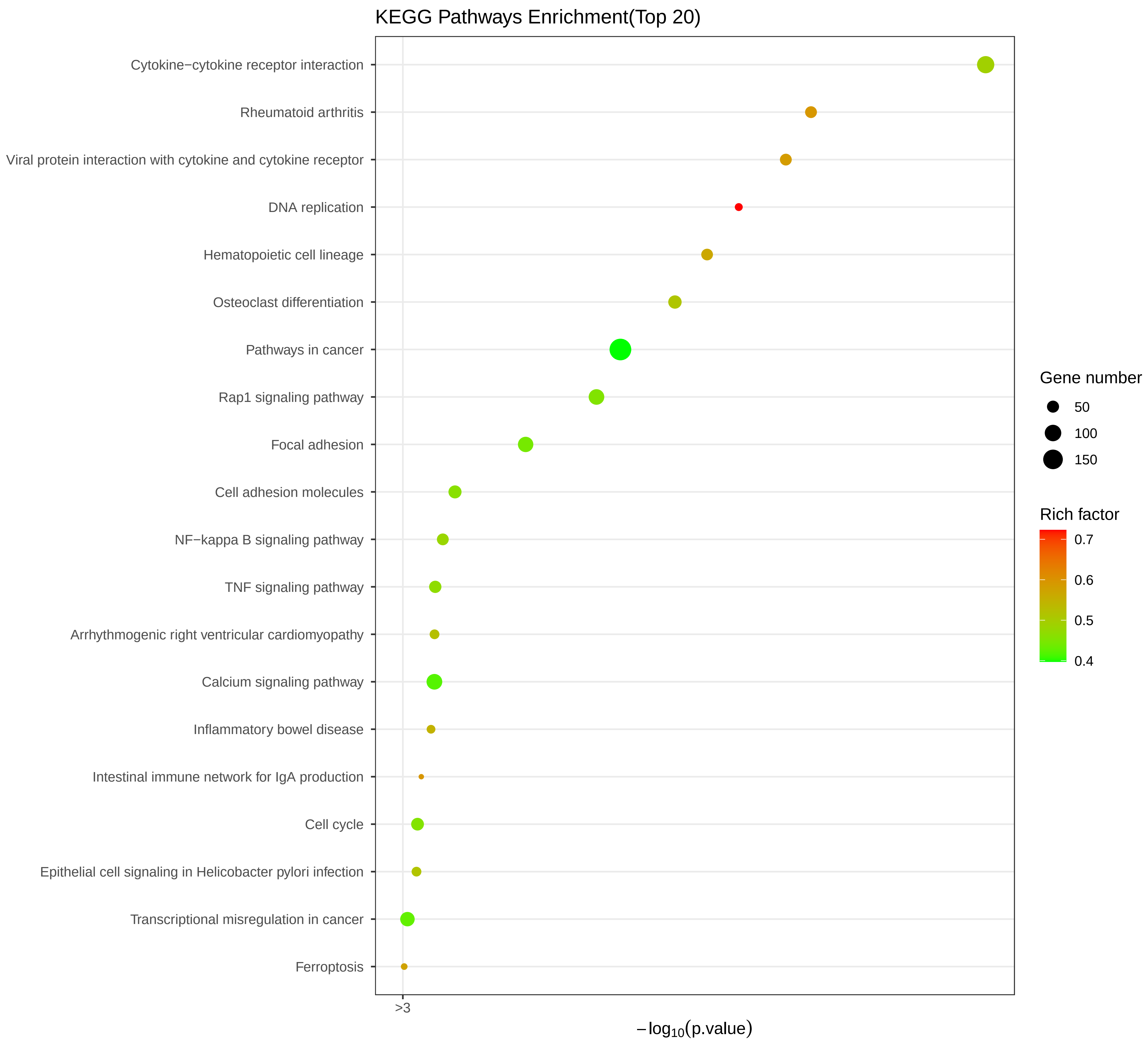

KEGG Pathways Enrichment(Top 20)
Cytokine−cytokine receptor interaction
Rheumatoid arthritis
Viral protein interaction with cytokine and cytokine receptor
DNA replication
Hematopoietic cell lineage
Osteoclast differentiation
Pathways in cancer
Rap1 signaling pathway
Focal adhesion
Cell adhesion molecules
NF−kappa B signaling pathway
TNF signaling pathway
Arrhythmogenic right ventricular cardiomyopathy
Calcium signaling pathway
Inflammatory bowel disease
Intestinal immune network for IgA production
Cell cycle
Epithelial cell signaling in Helicobacter pylori infection
Transcriptional misregulation in cancer
Ferroptosis
>3
$-\log_{10}(p.value)$
Gene number
50
100
150
Rich factor
0.7
0.6
0.5
0.4
